# Supplementary material for: Are carbon emissions trading and green financial instruments synergistic? -Comprehensive quantitative research based on content analysis
Source: PLoS One. 2024 Mar 7;19(3):e0298601. doi: 10.1371/journal.pone.0298601 (PMC10919601; doi:10.1371/journal.pone.0298601)
Supplement: S1 Data — (ZIP) [file pone.0298601.s001.zip › Data/地级市Z_F工作报告.zip!/║┌┴·╜¡/╣■╢√▒⌡╩╨/└┤╫╘╣1⁄2╓┌║┼í╛┬φ┐╦╩2╛▌═°í┐_2015.docx]

各位代表：

现在，我代表市人民政府向大会报告工作，请予审议，并请市政协各位委员提出意见。

一、2014年工作简要回顾

过去一年，是哈尔滨发展历程中困难多、任务重的一年，也是有效应对经济下行压力和复杂局面、进一步夯实打牢科学发展基础的一年。一年来，我们在省委、省政府和市委的正确领导下，紧紧依靠全市人民，深入贯彻落实党的十八届三中、四中全会和习近平总书记系列重要讲话精神，以及省委、省政府重大战略部署，牢牢把握稳中求进、改革创新总基调，坚定实施新战略，着力稳增长、促改革、调结构、惠民生，凝神聚力、真抓实干，全市经济社会发展迈出了坚实步伐。

——经济运行稳中向好。预计地区生产总值比上年增长7%左右，固定资产投资增长12.2%，实现社会消费品零售总额3070.9亿元，增长12.6%；居民消费价格上涨2%，低于全国控制上限；城镇新增就业13.7万人，登记失业率控制在3.72%以内，经济运行基本处于合理区间。全年完成地方公共财政预算收入423.5亿元，其中市本级233.9亿元，分别增长5.3%和10.7%。

——结构调整取得积极成效。粮食总产量首次突破350亿斤，实现历史性“十一连增”，连续三年成为全国产粮第一大市，五常、双城、巴彦、依兰、呼兰、尚志、宾县被评为全国产粮大县（市、区）。实现规模以上工业增加值849.4亿元，增长7.7%。服务业增加值占全市生产总值比重提高到54%。万元GDP综合能耗下降4.5%以上，二氧化硫和化学需氧量提前一年完成国家“十二五”减排任务。

——人民生活水平持续提高。预计城乡居民人均可支配收入分别达28800元和12100元，增长9%和12%。年初确定的10个方面、66个惠民行动项目件件落实，完成投资314.8亿元，全市人民在改革发展中得到了更多实惠。

——精神文明与民主法治建设得到加强。创建全国文明城市深得民心，市民文明素质明显提升，以朱占华、王东伟等为代表的一大批先进典型，成为新时期培育和践行社会主义核心价值观、引领哈尔滨文明风尚的时代楷模。自觉接受人大依法监督和政协民主监督，人大代表建议、政协提案办复率均达100%。荣获全国“六五”普法中期先进城市。创建全国最具安全感省会城市成效显著，刑事案件万人发案率连续三年保持省会城市最低，道路交通等安全生产事故大幅下降，信访案件办结率达95%。

各位代表，过去一年，我们按照市委十三届五次全会和市十四届人大三次会议部署，坚持从实际出发，咬定实施新战略、实现科学发展新跨越目标不动摇，突出重点，抓好落实，着力推进了以下六方面工作。

（一）立足全市长远发展，毫不动摇抓产业上项目。坚定不移地走产业强市、产业富民之路，持续加大产业项目建设力度，全年新开复工2000万元以上产业项目1299个，投入资金1262亿元，经济发展基础更加坚实。重点采取了以下措施：一是扩大招商上项目。通过央企对接会、民企龙江行、中俄博览会等系列招商活动，全年新签约各类产业项目398个，实现当年开工272个，到位资金163.9亿元。全市实际使用外资27.2亿美元、内资1754.4亿元，分别增长20.3%和32.7%。二是调整结构上项目。着力推进的200个重大技改项目和战略性新兴产业项目进展顺利，百威英博、哈轴轮毂、凯斯纽荷兰等一批产业项目陆续投产。重点推进的104个亿元以上现代服务业项目成效明显，华南城一期、海宁皮革城、远大群力购物中心等大型商业综合体投入运营，极大地提升了我市现代服务业品质。三是创新驱动上项目。通过与哈工大、中科院等大学大所携手创建的科技成果转化平台，集聚科技成果及产业化项目达3213个，实现落地转化1606个，进入产业化项目130个，为产业升级、经济转型注入了活力。四是建设园区上项目。持续扩大基础设施建设投入，把园区建设引导资金提高到6亿元，园区集聚力、吸引力和承载力明显增强。经开区新开复工产业项目100个，高新区入驻国内外研发创新机构达179家，利民开发区成为国家生物医药产业集聚发展试点，宾西开发区实现工业总产值增长25.7%，五常牛家工业园跨入省级开发区行列。五是金融支撑上项目。重点产业项目贷款余额达828.6亿元，当年新增113.1亿元，增长15.8%。市财政用于产业发展资金达24.4亿元，发行企业债券18亿元，企业信用融资担保服务中心担保额超过130亿元，哈创投成功与君丰资本等9支基金达成合作意向。均信担保等11家企业在新三板挂牌，哈尔滨银行、葵花药业、圣明节能分别在港交所、深交所、新加坡成功上市。六是创优环境上项目。持续加大涉企执法、“六项禁令”监督检查力度，共查处破坏经济发展环境相关案件135起，问责公职人员208人，极大地改善了经济发展环境。道里、道外、南岗、香坊和依兰县步入了全国投资潜力百强区和百强县，哈尔滨经济发展的基础更加牢固、思路更加清晰、措施更加有力。

（二）全力稳增长促发展，为调结构转方式提供了更大的回旋余地。沉着应对经济下行压力，把稳增长的着力点放在保存量、促增量、稳住经济基本面上，实现了经济社会持续健康发展。一是抢抓机遇争主动。认真研究消化国家支持东北振兴35条、全省稳增长65条政策意见，制定出台了我市工业稳增长新12条、扶持中小企业发展28条以及促进经济稳增长42条等一揽子政策措施，为全市经济发展注入了新动力。全市新增规模以上工业企业130户左右，非公经济增加值占GDP比重超过54%，香坊被列入国家老工业区搬迁改造试点。二是强化服务解难题。针对企业生产经营困难增多实际，我们创新了联审办公、综合调度、跟踪督办、运行监测预警等工作机制，帮助企业解决用电、用地、融资、运输等各类难题200多件，为企业发展创造了有利条件。三是挖掘潜能促增长。重点推动旅游文化时尚产业融合发展，万达文旅城、波塞冬海洋王国等大项目按计划推进，哈尔滨音乐厅、哈师大现代文化艺术产业园、犹太老会堂音乐厅建成使用，哈尔滨游客服务中心对外开放，“冰城夏都”品牌的影响力和吸引力显著增强，全年实现旅游总收入786.7亿元，增长17.7%。

（三）统筹城乡改革发展，持续壮大县域经济整体实力。积极抢抓两大平原现代农业综合配套改革机遇，深入实施富民强县战略，县域经济发展活力得到进一步释放。一是全力推进农村产权制度改革。率先在全省启动了土地确权登记颁证工作，目前已全部完成土地实测任务，配套建成各级产权流转交易平台1141个、土地纠纷仲裁机构12个，年内即可完成土地确权登记颁证工作，至少可以提前三年完成国家确定的改革任务。农村产权制度改革激发了农村市场活力，新增农民合作社438个，200亩以上土地适度规模经营面积扩大到400万亩，比农村土地确权前增加169万亩，是近十年来增速最快的一年。二是深入实施产业强县工程。遵循以资源换产业、以市场换项目的思路，招大引强上项目，新建续建各类产业项目339个，其中，亿元以上项目220个，完成投资385亿元，是上年的1.2倍；127个产业项目陆续建成投产，县域规模以上工业总产值增长了20%左右。三是加快发展现代农业。重点抓住水利、农机、科技三条主线，提高农业综合生产能力，现代农业示范园区（带）发展到218个，核心区面积突破1000万亩；新建扩建规模化养殖场（区）162个，肉蛋奶产量首次突破300万吨。四是扎实推进农村城镇化。重点推进了13个整县整乡城镇化试点和80个新农村示范村建设，撤并了82个村屯，1.8万农民实现了向城镇和新型农村社区有序转移，被国务院列为全国首批新型城镇化综合试点城市。新建农村饮水安全工程398处，创建省级生态县（市）2个、生态乡镇12个、生态村293个，木兰、方正等9个城关镇污水处理厂投入运营，33个贫困村实现整村脱贫。新建农村公路高等级路面402公里，改造危桥167座，木兰松花江公路大桥开工建设，城乡一体化发展进程明显加快。

（四）以创建全国文明城市为牵动，着力为百姓打造幸福宜居美好家园。始终牢记对全市人民做出的庄严承诺，持续加大规划建设管理力度，城市功能和形象品位大幅提升。一是突出城市规划引领。城市总体规划修改通过市人大审议并上报省政府，大松北地区等十大方面规划编制完成，既有控详规划实现滚动修编，城市开发建设更加规范有序。双城撤县设区为城市拓展了更大发展空间，哈西新区、哈东现代物流产业带成为新城崛起的新样板。二是完善城市交通体系。新建改造道路90条、桥涵37座，新平北桥、河松跨河桥建成使用，南马路、哈双南路竣工通车，哈齐客专全线贯通，哈西公路客运站投入运营，地铁安全载客超过6700万人次，地铁1号线三期、2号线一期和3号线建设顺利启动。新建停车场21处，新增停车泊位1.5万个，新建公交首末站10处，新增大容量环保公交车600台，“公交都市”创建经验得到国家充分肯定和推广。三是加强源网设施建设。新建改造水电气热管网1120公里，建设各类源网厂站33座，道里、南岗核心区域配电网改造全面完成，“智慧管网”地理信息系统建成使用，城市运行保障水平得到较大提升；拆并改造分散燃煤小锅炉803台，新增集中供热能力1500万平方米，居民供热投诉率下降18.8%。四是提高城市管理水平。重点落实长效管理机制，持续加大环境卫生、交通“三乱”等专项整治力度，创建环境卫生示范街区165个，纠正交通违法行为556万件。坚持城市建设拆违先行，对违章建筑一律零容忍、零补偿，累计拆除违章建筑2万多处、近800万平方米，至少为国家减少经济损失300亿元。持续提高环卫作业能力，主城区垃圾密闭化运输率达100%，主干道路机械化清扫率、水冲洗率和生活垃圾无害化处理率分别提高到70%、90%和85%以上。坚定实施绿色清冰雪，主干街路实现即下即清，背街背巷、居民庭院雪停即清，居民出行更加方便，冰雪城市特色魅力更加凸显。在2014年香港中国城市竞争力研究会发布的中国最干净城市排行榜中，我市位列全国内陆城市第一名。五是改善城市生态环境。实施污染减排项目101个，淘汰黄标车3.5万辆，完成既有建筑节能改造318万平方米。成高子镇、团结镇污水处理厂实现通水调试，日处理650吨污泥项目建成投用，城市污水集中处理率提高到88%，松花江干流水质保持功能区划标准。完成人工造林10万亩，城区新植树木50万株、新增绿地106公顷，哈尔滨变得越来越干净、越来越秀美、越来越有品位。

（五）坚持向改革要动力向开放要活力，加快构建统一开放、竞争有序的市场新体制。主要是突出问题导向，下功夫破除体制机制障碍。一是创新行政审批制度。在全国有立法权城市中，率先取消了市级自设的所有行政审批事项，国家和省下放我市行政审批事项全部分级下放到位。重新编制了覆盖建设项目审批33种情形的27个审批流程，审批时限平均压缩50%，办理最快的“招拍挂”工业仓储类建设项目只需23个工作日，实现了“一口受理、一表登记、一次审查、一网流转、一次发证”。商事制度改革激发了全民创业创新活力，全市新登记注册市场主体10.2万户，是上年的1.2倍。二是健全市场监管体制。率先在副省级城市中实现了工商、质监、物价“三局合一”，组建了市场监督管理局，整合食品安全相关职能，成立了新的食品药品监督管理局，并将区县（市）市场监管机构下放属地管理，有效解决了市场监管职能交叉、权责不清、监管缺失等突出问题。率先在全国开通了联合征信平台，启动失信人乘机、出国等联合惩戒措施，市场诚信环境得到较大改善。三是强化财税管理。把部门全部收支纳入预算管理，市级政府、部门及“三公”经费预算全面向社会公开，财政预算绩效管理引入第三方评价机制，市级到期政府性债务如期偿还。“营改增”试点范围扩大，取消、停征24项行政事业性收费和政府性基金，减轻企业负担16.8亿元。四是深化国资国企改革。市属国有企业集团全部派驻监事会，并面向全国选聘9名企业高管。新组建的市政、交通、文旅、住建、供水、排水等市属国有企业集团作用发挥越来越突出。在哈央企“三供一业”分离移交工作全面完成。哈轴制造公司合资重组取得实质性进展。成功拍卖地铁1号线站内广告经营权4.51亿元，高于起拍价达7000万元。重新界定国企功能，完善分类考核指标体系，初步形成定位清晰、权责明确的国资管理新体制。五是完善对俄开放功能。综合保税区启动联审程序，临空经济区总体规划上报国家待批，全国首个大飞机拆解项目正式签约落地。新开通3条对俄客货混载和货运包机航线。对俄金融合作取得新进展，中俄电商跨境在线支付平台和边境物流仓储中心建成运营，成功引进亚马逊、敦煌网等一批知名跨境电商企业，对俄邮政小包裹发运量占全国30%以上，实现过货量2274吨、销售额2.2亿美元，超过“北、上、广”，居全国第一位。

（六）顺应人民群众期盼，着力解决群众最关心最直接最现实利益问题，不断满足全市人民日益增长的物质文化需要。一是持续改善居民住房条件。开工建设各类保障房2.4万套、竣工2.2万套，超额完成国家和省年度计划指标；为1.6万户困难群众提供廉租房保障；改造农村泥草房6万户、359万平方米；为历史遗留的8.7万户居民提供了房屋产权办证条件；道外新一地区6370户内涝居民全部得到搬迁安置。物业管理年活动成效明显，维修老旧住宅798万平方米，创建市级以上物业管理示范小区150个，清除不合格物业企业12家，老旧小区物业管理实现全覆盖。二是不断提高社会保障能力。实施更加积极的创业带动就业政策，创建了中国·哈尔滨人力资源中心，成功举办了第三届创业就业博览会，为应届高校毕业生、城镇就业困难人员、农村转移劳动力创业就业提供了更多援助和支持。持续扩大各类社会保险覆盖面，1.2万被征地农民养老保险遗留问题得到解决，市本级企业退休人员养老金人均月增172元；新建社区日间照料服务中心和农村居家养老服务站677个，新增养老床位5000张，成功获批全国养老服务业综合改革试点城市；城乡居民大病保险和县级公立医院改革试点启动实施，分级诊疗模式初步建立，市第一医院内部管理成效明显，新农合人均筹资标准由350元提高到390元。三是强化基本公共服务。创建义务教育标准化学校和素质教育特色（示范）学校57所，首创的冰雪运动学校成为国内体教联合办学典范，幼儿师范高等专科学校新校区投入使用，学前教育进入全国先进行列。成功举办了第32届哈尔滨之夏音乐会，哈尔滨俄罗斯音乐文化交流中心正式成立，南岗创建的国家公共文化服务体系示范区通过中期督查。全市新建千米以上城市社区和农村示范社区20个，成为全国和谐社区建设示范城市。

各位代表，过去一年取得的成绩来之不易。这是省委、省政府和市委正确领导的结果，是全市各级领导班子团结奋进、扎实工作的结果，是人大代表、政协委员及中省直单位和社会各界鼎力支持、全力配合的结果，是全市人民拼搏进取、艰苦奋斗的结果。在此，我代表市人民政府，向所有关心支持哈尔滨事业发展的同志们、朋友们，向广大驻哈解放军指战员、武警官兵，致以崇高的敬意和衷心的感谢！

在肯定成绩的同时，更要清醒地认识到，我们在发展中还存在不少困难和问题：主要是发展速度慢，产业规模小、实力弱的现状还没有得到有效改善，目前我市仍是唯一一个规模以上工业增加值没有过千亿元的副省级城市；经济发展质量和效益还不高，结构调整步伐还不够快，财政和城乡居民收入及社会保障标准在同类城市中仍处于较低水平；市场化程度偏低，利用资本市场直接融资的能力还不强，项目“招拍挂”市场竞争不够充分；高端人才比较匮乏，项目运营、资本运作、企业管理等领域大多缺少领军人物；经济开放度偏低，对经济增长的拉动力十分有限；政府职能转变仍有很大空间，体制机制弊端亟待破除，少数公务人员作风飘浮、能力不足，还存在为官不为、庸政懒政倾向，腐败行为和损害群众利益问题仍时有发生。对此，我们要高度重视，始终坚持用发展的手段解决前进中的问题，用改革创新的办法破解发展中的障碍，努力在克服困难、奋发进取中开拓更加广阔的发展前景！

二、2015年主要任务

2015年是全面深化改革的关键之年，是全面推进依法治市的开局之年，也是全面完成“十二五”规划的收官之年。做好今年工作，意义重大、影响深远。

新的一年，宏观经济形势正在发生深刻变化，经济发展已经进入新常态，对此，中央和省委经济工作会议做出了战略判断和深刻分析，无论是全国还是全省经济发展都已进入了一个以速度换挡、结构优化、动力转换为基本特征的新常态，市场需求、生产能力和产业组织方式、生产要素相对优势、市场竞争特点、资源配置模式等都已发生趋势性变化，经济发展正在向形态更高级、分工更复杂、结构更合理的阶段演化。哈尔滨作为后发地区、老工业基地城市，经济发展新常态阶段性特征更为明显。

一是新常态带来新变化。经济增长正从高速增长转向中速增长；经济发展方式正从“原字号”、粗放式，向以要素驱动为基础、创新驱动和投资拉动并重转变；产业结构正从以传统产业为主，向战略性新兴产业和现代服务业拓展；发展动力正从传统体制机制拖累负重前行，向重塑改革开放新动力迈进。这些具有历史性和转折性的新趋势、新变化，既是我们促进经济增长的新动力，更是哈尔滨未来发展必须遵循的新方向。

二是新常态带来新挑战。历经三十多年高强度大规模开发建设，我市传统产业包括低档次商贸服务业投资相对饱和，投资拉动作用开始减弱；过去那种模仿型排浪式消费阶段已基本结束，外部需求不振的状况短时间内难以改变，扩内需促增长面临着许多新考验；另外，我市传统优势产业大多已由“成熟期”进入“衰退期”，一些“国字号”大企业在市场竞争中举步维艰，有的发展优势变成了巨大包袱，企业依赖税收和土地等优惠政策形成竞争优势、外资超国民待遇的方式已经难以为继，特别是资源环境刚性约束越来越强，迫切需要我们在转变经济发展方式、提升发展质量上迈出更大步伐。

三是新常态带来新机遇。国家和省相继推出了一系列稳增长、促改革、调结构、惠民生激励政策，包括支持东北振兴35条等，很多都是为我市量身定制的特殊政策，今年必将持续发力，有利于我们在深化改革、扩大开放、创新发展上获得更大的支持。省委、省政府在实施“五大规划”、建设“十大重点产业”过程中，更加注重发挥省会城市龙头带动作用，我们着力打造的绿色食品、汽车、机器人、燃气轮机以及旅游文化时尚产业，越来越受到省委、省政府的关注和支持，为我们实现率先发展、跨越赶超创造了更加有利条件。只要我们抓住这些机遇、用好这些机遇，乘势而上、顺势而为，就一定能够在新常态下实现经济社会更好更快发展。

四是应对新常态、实现新发展，我们既有基础、更有信心。改革开放三十多年的生动实践，历届市委、市政府打下的坚实基础，让哈尔滨积蓄了巨大的发展潜能。特别是通过坚定实施新战略和大规模基础设施建设，累计新建改造各类道路661条、桥涵193座，不仅拉开了城市骨架，完备了城市功能，更奠定了“一江居中、两岸繁荣”的城市发展格局，广大投资者对我们这座城市越来越向往、越来越有信心。通过持续抓产业上项目，三年累计开工建设2000万元以上项目2140个，投入产业资金达3802.2亿元，已有1080个项目建成投产，新增产值超过500亿元、利税94.6亿元，产业带动作用明显增强。通过统筹城乡一体化发展，县域经济综合实力显著增强，贡献率已由2011年的30.1%提高到52.4%。通过连续开展以行政问责为核心的政风建设年活动，从严整治涉企“四乱”行为，经济发展环境明显改善，人心思干、人心思进、人心思变已经成为时代主流，全市上下聚精会神谋发展、齐心协力促振兴的大好局面已初步形成。只要我们发挥好这些比较优势，最大限度地释放发展潜能，就一定能够在大有作为中开创更加美好的未来！

按照市委十三届七次全会要求，政府工作的指导思想是：以党的十八大、十八届三中、四中全会、中央经济工作会议和习近平总书记系列重要讲话精神为指导，认真贯彻落实省委、省政府部署要求，坚定实施新战略，奋力实现新跨越，坚持稳中求进工作总基调，以改革开放创新为动力，以全面依法治市为保障，以提高经济发展质量和效益为中心，把转方式调结构放到更加突出的重要位置，着力做好稳增长、促改革、调结构、惠民生等工作，努力完成“十二五”规划，科学描绘“十三五”发展蓝图，奋力开创全面建成小康社会新局面。

经济社会发展的主要目标是：地区生产总值增长7%左右；固定资产投资增长7%左右；规模以上工业增加值增长7%左右；社会消费品零售总额增长12%左右；地方公共财政预算收入增长3%左右；市属外贸进出口总额增长5%左右；城镇居民人均可支配收入增长9%以上，农村居民人均可支配收入增长10%以上；城镇居民登记失业率控制在4.3%以内；万元GDP综合能耗下降2.46%以上。为实现上述思路目标，我们要着重做好以下六项工作：

（一）继续坚定抓产业稳增长，奋力构筑经济转型升级新优势。抓产业既是稳增长促发展的当务之急，也是调结构增后劲的治本之策，我们要把保持经济平稳健康发展的着力点放在产业项目建设上，调整存量、做优增量、做大总量，全年重点建设2000万元以上产业项目要超过1000个，完成投资要超过800亿元。

工业是哈尔滨的立市之基、强市之本，工业稳则全局稳。我们要从盘活既有存量资源入手，坚持存量扩张式、技术升级式、合资合作式发展思路，加快改造提升传统优势产业，培育壮大战略性新兴产业，持续推动产业层次向中高端迈进。立足打造千亿级装备制造产业，继续实施工业“双百工程”，集中力量抓好哈飞汽车合资合作等重大技改项目100项，中船重工中小型燃气轮机等战略性新兴产业项目100项，新增规模以上工业企业100户。立足壮大千亿级食品工业，进一步叫响绿色食品品牌，集中力量抓好畜产品、粮油制品、果蔬饮料加工等一批重点项目。立足发展千亿级云计算、物联网、电子商务等信息产业，集中力量抓好阿里巴巴哈尔滨平台等大项目建设，完善中国云谷服务功能，努力在国家电子商务示范城市建设上取得更大进展。

现代服务业既是调结构的“牵引器”，也是稳增长的重要支撑。我们要着力发展现代金融、物流、信息、商务会展、科技研发与技术创新等生产性服务业，重点做好哈尔滨银行60亿元金融债券发行工作，支持中飞新技术、威帝电子、奥瑞德等更多企业上市融资，力争区域股权交易市场落户我市，切实增强金融服务实体经济能力。加快推进华南城、传化智能公路港、国际农业博览中心等大项目建设，进一步提升商贸物流会展业发展质量。突出旅游文化时尚产业融合发展，深度整合历史建筑、高雅艺术、时尚演出、景区景点以及冰雪、湿地、森林等优势资源，重点办好俄罗斯顶级艺术家油画作品常设展，建好哈尔滨国际油画艺术品交易中心，抓好万达文旅城、波塞冬海洋王国等大项目建设，确保哈尔滨大剧院年内建成使用。高质量办好冰雪节、金上京建都900年、“迷人的哈尔滨之夏”等系列活动，全年实现旅游总收入增长15%以上。

在传统产业增长动力减弱的情况下，必须更多地依靠科技创新培育产业竞争新优势。进一步建立完善政、产、学、研协同创新机制，支持科技成果转化平台、省工研院、机器人产业技术研究院建设发展，创办以农大为主，商大、黑大、省市农科院参股的食品产业技术研究院，填补科技成果转化及产业化的中间地带，全年实现科技成果转化落地项目300项。完善科技创新城配套功能，深化与中关村合作，更多地引进中科院、中国工程院重大技术成果落地产业化，努力争创国家级自主创新示范区。鼓励支持科技人员领办、参办科技型小微企业，形成大众创业、万众创新的浓厚氛围。发挥创业投资引导资金作用，大力引进和培育创业投资、风险投资等私募基金，不断开辟科技成果产业化融资新途径。

园区是产业集聚的平台，也是牵动全市经济发展的重要增长极。今年，园区发展专项资金提高到8亿元，产业扶持资金新增20亿元，重点支持基础设施建设和主导产业发展。经开区重点以发展高端装备、绿色食品和电子信息产业为方向，开复工重大产业项目至少40项，战略性新兴产业比重要超过20%。高新区重点以科技创新及产业化为引领，加快推进22个产业项目建设，确保中航科工钛合金等18个项目建成投用。利民开发区重点以生物医药为依托，突出抓好20个产业项目建设，确保天宏药业等15个项目建成投产。宾西开发区重点以现代包装和农副产品加工为主导，开工建设至少10个产业项目，确保天地丰肥业等5个项目竣工投产。力争双城开发区晋升为国家级开发区，巴彦、通河、延寿晋升为省级开发区。

招商引资是引进增量、做优存量的重要抓手，也是实现弯道超车、跨越赶超的有效途径。必须坚持规模与质量并重，进一步完善产业大招商目标责任体系和重大产业项目引进评估机制，全力开展专业招商、板块招商、以商招商、产业链招商，更多地引进有助于优化产业结构、促进产业集群、打造特色专业园区的大项目好项目。全年实际使用外资和内资分别增长10%以上。

产业建设既不是一时之功，更不能一劳永逸，只要我们咬定青山不放松，一年接着一年抓、一任接着一任干，就一定会在日趋激烈的市场竞争中赢得主动、创造未来。

（二）继续推进富民强县战略，不断使县域经济在加快发展中释放更大潜能。广袤的农村腹地和富集的农业资源，是实现哈尔滨加快发展的重要战略支撑。我们要按照两大平原现代农业综合配套改革要求，着力在转变农业发展方式、统筹城乡发展上取得更大进展。

深化农村各项改革。坚持公平公正公开原则，妥善处理各类土地矛盾，年内基本完成承包地、宅基地、集体建设用地确权登记颁证工作，实现市、区县（市）、乡镇三级产权交易服务平台联网运营。抓好香坊、方正农村集体经营性建设用地流转试点，有序推进农村集体建设用地入市改革。积极鼓励发展种养大户、家庭农场、农民合作社和多种形式的适度规模经营，全年实现土地流转面积700万亩，200亩以上土地适度规模经营面积扩大到600万亩。率先在全省启动五常、双城、方正新型农业社会化服务试点，努力为现代农业生产经营提供更有效的服务。

深入推进产业强县工程。重点围绕建设八大产业、三大养殖加工板块和十大特色产业基地，加快提升以绿色食品为重点的农业标准化生产和产业化经营水平，促进一二三产业融合发展。重点抓好宾西牛业、双城雀巢等50户龙头企业，着力打造尚志山产品、依兰煤化工、巴彦皮草皮革加工等特色产业园，加快“巴木通方依”区域产业一体化进程，力争县域工业总产值占全市工业比重超过43%。

大力发展现代农业。突出数量质量效益并重，持续提升农业综合生产能力，新建现代农业示范园区（带）32个，粮食总产稳定在350亿斤以上，农业综合机械化率提高到90%。大兴农田水利，重点抓好通河二甲沟水库、木兰柳河提水泵站等10个项目建设。集约发展畜牧业，新改扩建标准化养殖场（区）100个，肉蛋奶总产量保持在300万吨以上。

突出抓好城镇化和新农村建设。重点以国家新型城镇化综合试点为牵动，编制新型城镇化总体规划，抓好阿城国家“多规合一”试点，有序推进农业转移人口市民化，着力构建统筹发展、功能完备、特色鲜明的现代城镇体系。继续抓好木兰松花江公路大桥建设，完成哈同公路钢丝厂至二龙山段改扩建工程，新建农村公路高等级路面300公里，交工通车200公里，改造危桥100座。坚持“物的新农村”和“人的新农村”建设齐头并进，启动建设新型农村社区试点100个，新建农村饮水安全工程332处，加快推进68个贫困村整体脱贫。抓好农民工技能培训，让更多的进城务工农民掌握一技之长。

县域是我市未来发展大有可为的广阔空间。我们要比以往任何时候都更加重视和支持县域经济、推动“三农”工作，努力让县域经济在竞相发展中释放潜能、迸发出更大的发展活力。

（三）继续加强城市规划建设管理，努力让城市更干净更文明更有魅力。围绕建设现代化国际化城市目标，我们要始终保持创建全国文明城市的标准和力度，持续提升城市规划建设管理水平。

进一步搞好城市规划设计。发挥规划引领开发建设作用，精心编制彰显城市特色、促进产城融合、创造宜居环境等城乡发展十大支撑性规划，实现城市总体规划、专项规划、城市设计和详细规划有序衔接，努力为城市健康发展提供有力支撑。

进一步完善城市载体功能。强化项目建设、资金筹措和科学管理，有序推进城市开发建设。全年新建改造城区道路90条、桥涵15座，畅通综保区、林大科技园等区域微循环路网，确保公滨路与四环路互通立交桥、阳明滩北部联络线等路桥竣工通车。继续推进地铁1号线三期、2号线一期、3号线一期和二期工程、哈南停车场及出入场线建设。配合哈站改造，完成地工街、一面街地道桥等一批道路疏解工程。支持哈佳、哈牡客运专线、滨北公铁两用桥及太平机场扩建等重大项目建设。统筹推进城区老旧管网改造，完善智慧管网信息系统，新建改造各类源网厂站23座、管网658公里。启动建设松花江水源供水工程，消除城市单一水源供水安全隐患。深入开展“公交都市”创建活动，新建停车场19处，新增停车泊位8000个，开辟调整公交线路20条，新增更新公交车辆600台，启动建设1座大型公交枢纽站、3处公交首末站，努力让百姓出行更方便，城市运行更有保障。

进一步提高城市管理水平。全面落实城市管理精细化、长效化机制，深入整治背街背巷、居民庭院、市场摊区、露天烧烤、基建工地、城乡结合部等脏乱差环境，持续提高机械化作业能力，城区机械化清扫和清冰雪率均提高到80%以上。始终保持拆违控违高压态势，从严整治交通“三乱”、船舶码头及道路货物运输市场等城市乱象，确保城市面貌实现更大改观。继续开展物业管理年活动，坚决取缔不合格物业企业，新增物业管理面积600万平方米，维修老旧住宅700万平方米以上，创建市级以上示范物业小区100个，努力让百姓生活得更舒心、更满意、更有幸福感。

进一步加强生态环境保护。坚持源头严防、过程严管、后果严惩、标本兼治，推进节能减排和生态保护。集中整治大气污染，着力抓好秸秆固化燃料加工和原料收储基地建设；继续实施供热燃煤锅炉改造，重点推进剩余的10吨以下小锅炉撤并，同步启动20吨以下燃煤锅炉撤并工作；全市淘汰黄标车4.2万辆，确保空气污染源头治理取得较大进展。积极推进生态保护与修复，实行最严格的水资源管理，突出抓好松花江流域治污项目和马家沟水源工程建设，全年完成人工造林7万亩，城区新植树木20万株、新增绿地60公顷，实施农村生态治理项目71个，治理水土流失36万亩，努力建设生态文明的美好家园。

（四）继续深化经济体制改革，全力打造有利于激发市场活力的新体制。推动经济平稳健康发展，要求我们必须发挥经济体制改革的牵动作用，力争使改革红利更多地转化为发展新动力。

行政管理体制改革是经济体制改革的突破口，也是现行条件下提升投资吸引力的关键所在。今年我们要继续抓好网上审批大厅、行政审批云平台系统建设，进一步优化审批流程。扎实做好审批事项“接管放”，推行审批项目清单管理，强化事中事后监管，真正把该放的事权放到底、该管的事权管到位。加大中介机构规范清理力度，重点推进中介机构与行政机关或挂靠事业单位脱钩改制，重新编制中介服务项目目录，并向社会公开，凡是没有规定自设的服务项目一律取消，凡是中介机构承担的审批部门事项一律收回，凡是企业需要中介机构服务的一律不得指定。按照简政放权、放管结合新要求，扎实推进政府机构改革，科学配置内设机构及职责，有效解决权界不清、职能交叉、政出多门、重复管理等问题。

国资国企改革是经济体制改革的重头戏。我们要制定出台深化国资国企改革实施意见，重点推进国有资本授权经营体制改革，实施经营性国有资产跨行业跨部门整合，逐步实现政府公共管理职能与国有资产出资人职能分离。调整优化国有经济布局，发展混合所有制经济，启动工投集团所属28户竞争性企业产权多元化和股份制改革试点，确保马迭尔集团、麒麟集团、旭丰农机公司合资重组取得实质性进展。进一步建立健全现代企业制度，完善国有企业市场化选聘职业经理人机制，力争国有独资企业集团外部董事和监事实现全覆盖。

财税和投融资体制改革是经济体制改革的重点。我们要贯彻落实好新修订的《预算法》，调整完善预算编制方式，建立跨年度预算平衡机制，继续扩大一级政府财政预算绩效管理和第三方绩效评价试点范围。健全政府举债新机制，把政府债务分门别类纳入全口径预算管理，有效防范和化解政府性债务风险。推动投融资结合，发挥财政资金放大和撬动作用，探索政府与社会资本合作新途径，更多地引进社会资本参与基本公共服务项目建设。

非公经济是激发市场活力和社会创造力的重要引擎。我们要深入实施中小微企业成长工程，进一步落实中小企业扶持政策，培育壮大一批民营企业集团，支持私营企业建立现代企业制度。抓好民营经济发展改革省级试点，放宽民间资本进入行业和领域，鼓励各类社会资本参与国有企业改制重组，努力为非公经济搭建创业创富、大显身手的广阔舞台。

（五）继续扩大沿边开发开放优势，着力在打造对俄合作中心城市上迈出更大步伐。以哈尔滨为中心，构建“中蒙俄经济走廊”龙江陆海丝绸之路经济带，是中央和省委推出的重大战略举措。我们必须主动作为，率先发力，加快构建多层次、宽领域、跨境互动对俄开放合作新格局。

着力完善基础设施互联互通。重点依托美国太平洋国际物流公司和四川航空公司，力争年内开通哈尔滨至旧金山客货运航线，同时谋划开通哈尔滨至莫斯科货运专线。加快哈尔滨内陆港搬迁至铁路集装箱中心站，确保年底实现对俄集装箱整车发送。积极拓展与绥芬河、海参崴和纳霍德卡陆海联运合作，加快构建面向俄罗斯、辐射东北亚、连接北美地区的国际货运大通道。

积极吸引国内外产业向哈尔滨集聚。重点抓好综合保税区规划建设和招商工作，继续跟踪落实哈飞复合材料、俄罗斯西格玛小型飞机等一批支撑项目，同步推进相关路网等基础设施建设，力争年底综保区一期达到封关运营条件。抓好临空经济区征地拆迁、产业引进和配套设施建设，确保大飞机拆解、绿地空港城等重大项目落地开工。依托国家政策支持，启动松江新区申报工作。充分利用俄罗斯石油、天然气、宝玉石等资源，规划建设对俄进出口加工产业园区，打造跨境资源综合利用产业链。

加快推动对俄经贸合作转型升级。重点抓好跨境电商物流仓储基地、华南城对俄大厦、华夏天元对俄物流等项目建设，力争对俄小包裹发运量占全国半壁江山，吸引更多的对俄出口加工制造企业落户哈尔滨。持续推动双方大宗商品交易，抓好100万吨俄油加工、诺林化工等项目，扩大机电设备、农产品、轻工产品对俄出口，全年实现对俄进出口贸易额增长10%以上。

大力促进对俄多领域交流。注重优势互补，密切友城往来，着力深化对俄文化、旅游、科技、金融等多领域合作，高质量办好哈尔滨中国·俄罗斯文化艺术活动周和“哈科会”明斯克专场，积极落实太平国际机场外国游客72小时过境免签政策，加快建设面向俄罗斯及东北亚区域金融服务中心，为扩大对外交流合作创造更加有利的条件。

（六）继续坚持为群众办实事办好事，最大限度让广大人民群众在改革发展中得到更多实惠。今年重点实施10个方面、69个惠民行动项目，完成投资260亿元，进一步织密织牢民生保障安全网。

就业乃民生之本、住房是民生之要，我们要创造更加优良的创业就业政策环境，努力为高校毕业生、农村转移劳动力、城镇困难就业人员等重点群体创造更多的创业就业机会。全年新增城镇就业11.7万人，转移农村劳动力148万人。发挥人才引进资金作用，吸引更多的高端人才在哈尔滨工作和创业。继续加大保障性安居工程建设力度，开工建设各类保障房1.3万套、竣工1.6万套，为1.4万户低收入家庭提供住房保障；改造农村泥草房5万户、283万平方米，努力让广大群众在安居乐业中生活得更体面、更有尊严。

教育关乎孩子成长、医疗关乎家庭幸福，我们要始终把教育事业摆在优先发展位置，坚持以促进教育公平、提高教育质量为主线，继续深化教育综合配套改革，积极促进义务教育均衡发展，创建义务教育标准化学校、素质教育特色（示范）学校32所，新建改造中小学校36所。积极稳妥改革中等学校招生考试制度，鼓励发展民办教育。进一步落实困难学生资助政策，努力让每一个困难家庭孩子都能上得起学、上好学。加快职业教育园区建设，密切校企合作，构建以就业为导向的现代职业教育体系，努力为我市产业发展培养更多的适用型人才。为进一步缓解群众看病难、看病贵，加快整合优质医疗资源，抓好分级诊疗、双向转诊体系和区域医疗机构联合体建设，推进医生多点执业，让群众能够就近享受更多的优质医疗服务。扎实推进医药卫生体制改革，全面实施城乡居民大病保险制度，二次报销比例不低于50%，最高达70%，新农合人均筹资标准由390元提高到450元；把县级公立医院综合改革试点扩大到每个县（市），实行药品零差率销售。完善公共卫生服务体系，人均基本公共卫生服务经费由35元提高到40元，免费为城乡居民提供更多的基本公共卫生服务。

社保和养老事关每个人的切身利益，我们要持续扩大社会养老保险覆盖面，重点做好基本养老金调待、新农保与城居保两险合并等工作。针对我市困难群体缴费难的实际，通过政府贴息、银行贷款垫付等方式，为困难群体缴纳养老金。完善失地农民生活保障制度，妥善解决被征地农民养老保险问题。适时启动城乡低保标准动态调整机制，切实保障困难群众基本生活。为更好地应对人口老龄化，突出抓好养老服务业综合改革试点，推行“医养结合”养老服务模式，鼓励社会力量兴办养老健康产业和养老服务机构，全年新建日间照料服务中心430个、农村居家养老服务站224个，新增养老床位5000张，区县（市）综合性社会福利中心实现全覆盖。

文化是人民的精神家园，安全稳定是群众安居乐业的重要保障，我们要深入实施文化惠民工程，继续推进市、区、街道（社区）和县、乡（镇）、村两个三级公共文化设施建设，让城乡居民享有更多的公共文化服务。持续提升文化发展水平，精心打造百年交响乐团、松雷音乐剧团等艺术团体，抓好犹太老会堂室内音乐演出、“万达秀”旅游驻场演出、音乐剧驻点演出等文化产品，更好地丰富广大人民群众业余文化生活。切实加强历史名城保护，建设中东铁路公园，继续做好“731”和金上京申遗工作，“731”遗址新馆确保9月3日前对外开放。积极培育和践行社会主义核心价值观，深入开展文明单位、文明村镇、文明行业、文明社区等群众性精神文明创建活动，努力塑造文明哈尔滨新形象。积极开展全民健身活动，规划建设哈尔滨奥体中心，加快打造城区10分钟健身圈，村级健身设施实现全覆盖。深入实施“强基工程”，持续提升社会服务能力，新建千米以上城市社区、农村示范社区20个以上，创建四星级以上和谐社区120个。围绕打造最具安全感省会城市，进一步完善网格化、立体化社会治安防控体系，严厉打击各类违法犯罪行为。加强公共安全智能化、精细化管理，落实“党政同责、一岗双责”安全生产责任制，严格排查整治安全隐患，有效防止和坚决遏制各类重特大事故发生。加强食品药品安全监管，建立完善从生产加工到流通消费全程监管机制、社会共治制度和可追溯体系，确保百姓“舌尖上的安全”。加强重大决策社会稳定风险评估，依法解决信访问题、化解社会矛盾。继续做好民族宗教、妇女儿童、人口和计划生育、残疾人、外事侨务、国防建设、双拥及民兵预备役等工作。

三、加强政府自身建设

面对发展新常态、新特征、新要求，我们要进一步加强法治政府、创新政府、廉洁政府建设，切实增强政府的执行力和公信力，努力为全市人民提供更加优质高效的服务。

（一）坚持解放思想，着力推动改革创新。大力弘扬改革创新精神。鼓励和引导政府工作人员，坚定发展信心，保持昂扬锐气，始终以改革精神谋划发展，以统筹办法推动工作，以创新举措打破利益固化藩篱，着重在产业引进、重大基础设施建设、国有资产管理经营等方面提高市场化运作水平，在破除体制机制弊端中增动力、促转型、快发展。扎实推进改革创新实践。着力在老工业基地振兴、新型城镇化推进等方面先行先试，在发展混合所有制经济、构建现代企业制度等方面探索新路，真正让各项改革措施落到实处、抓出成效。努力提高改革创新能力。牢牢把握改革的规律性、阶段性特征，着力提高战略思维、辩证思维、底线思维能力，提高统揽全局、协调各方、应对复杂局面、合力创新突破的能力，有效解决改革中遇到的各种矛盾和问题，努力创造经得起人民和历史检验的改革发展新业绩。

（二）坚持依法行政，持续改善发展环境。牢固树立法治理念。坚持用法治思维法治方式推动改革发展，加快形成办事依法、遇事找法、解决问题用法、化解矛盾靠法的良好法治氛围。严格规范行政行为。坚持法定职责必须为、法无授权不可为，确保权力运行守边界、有约束、受监督。健全决策机制，把公众参与、专家论证、风险评估、合法性审查、集体讨论决定确定为重大行政决策法定程序，确保决策制度科学、程序正当、过程公开、责任明确。建立推行政府法律顾问、重大执法决定法制审核、重大决策终身责任追究制度以及责任倒查机制，使政府各项工作始终在法治轨道上运行。加大信息公开力度。重点推进决策公开、执行公开、服务公开、管理公开、结果公开，加快打造“阳光政府”。认真接受人大依法监督、政协民主监督和社会舆论监督，充分听取工、青、妇等人民团体意见建议。加快推进企业信用信息公开，大力整治失信违法、扰乱市场秩序等行为，让守信者畅通无阻，让失信者寸步难行。继续强化行政问责。重点整治一线科所队和基层专管员，坚决纠正吃拿卡要及不作为、乱作为、慢作为、“中梗阻”等行为，坚决克服庸懒散拖等不良风气，严厉查处破坏经济发展环境的人和事，切实打通服务企业和群众“最后一公里”。

（三）坚持转变作风，全力推进工作落实。深入贯彻落实中央和省、市委改进作风相关规定，驰而不息纠正“四风”，努力在转作风中提高政府工作效能。突出强化抓落实的责任。严格政府工作目标考核、绩效管理，建立健全纵向到底、横向到边责任推进机制，确保事事有人抓、件件有人管、项项能落实，真正把广大政府工作人员的全部精力都汇集到干事创业上，体现在保障改善民生上。始终保持抓落实的韧劲。特别是在产业发展、城市建设、民生改善等事关改革发展大局和群众切身利益的大事要事上，我们要保持定力、持续推进、一抓到底，以敢于担当、敢于承重、敢于碰硬的锐气，以踏石留印、抓铁有痕、苦干实干的作风，以一环紧着一环拧、一锤接着一锤敲的执着精神，奋力开创新局面、创造新业绩。不断提高抓落实的效率。对定下的任务目标雷厉风行、马上就办，对难得的政策机遇盯住不放、有效利用，对看准的创新举措大胆探索、全力推进，以先人一步、快人一拍的自觉行为，不断增强政府工作的执行力和创造力。

（四）坚持廉洁自律，努力做到干净干事。加强廉政教育。牢固树立正确的权力观、利益观和政绩观，自觉遵守廉政准则，始终严以修身、严以用权、严以律己，知所循、知所守、知所惧，切实做到防微杜渐、警钟长鸣。完善制度建设。深入落实“两个责任”，严格执行“一岗双责”、“一案三查”、“一问四责”和相关规定，不断增强制度约束力，真正做到用制度管权管事管人。严守纪律要求。坚持原则、恪守规矩，严守政治纪律、组织纪律、财经纪律、工作纪律和生活纪律，坚决破除一切“潜规”、“陋规”，确保说到做到、有纪必执、有违必查。加大监督力度。切实加强对行政审批、项目招投标、土地出让、政府采购、公共资源转让、国有资产产权交易等重点领域、关键环节的行政监察和审计监督，持续保持查处腐败高压态势，严厉查处顶风违纪行为，坚决遏制腐败蔓延势头，努力使所有公务人员廉洁奉公、勤勉尽责，真正当好人民公仆。

各位代表，人民赋予重托，拼搏创造未来！让我们紧密团结在以习近平同志为总书记的党中央周围，在省委、省政府和市委的正确领导下，紧紧依靠全市人民，团结一心，励精图治，奋发图强，为实现哈尔滨科学发展新跨越、谱写全市人民美好生活新篇章而努力奋斗！
